# *Study Protocol* effectiveness of a nutritional intervention based on encouraging the consumption of unprocessed and minimally processed foods and the practice of physical activities for appropriate weight gain in overweight, adult, pregnant women: a randomized controlled trial

**DOI:** 10.1186/s12884-019-2672-1

**Published:** 2020-01-07

**Authors:** Daniela Saes Sartorelli, Lívia Castro Crivellenti, Marina Garcia Manochio-Pina, Naiara Franco Baroni, Mariana Rinaldi Carvalho, Rosa Wanda Diez-Garcia, Laércio Joel Franco

**Affiliations:** 1 Department of Social Medicine, Ribeirão Preto Medical School, University of São Paulo, Brazil, Avenida Bandeirantes, 3900, Ribeirão Preto, SP 14049-900 Brazil; 2 Graduate Program of Public Health, Ribeirão Preto Medical School, University of São Paulo, Brazil, Avenida Bandeirantes, 3900, Ribeirão Preto, SP 14049-900 Brazil; 3 Program in Health Promotion, University of Franca, Avenida Dr. Armando Salles Oliveira, 201, Franca, SP 14404-600 Brazil; 4 Department of Health Sciences, Ribeirão Preto Medical School, University of São Paulo, Brazil, Avenida Bandeirantes, 3900, Ribeirão Preto, SP 14049-900 Brazil

**Keywords:** Pregnancy, Randomized clinical trial, Protocol, Gestational weight gain, Food processing, NOVA classification, Overweight, Lifestyle intervention, Newborn, Adiposity

## Abstract

**Background:**

Evidence from observational studies suggests that a greater intake of ultra-processed foods during pregnancy is associated with a higher chance of obesity, increased gestational weight gain, and neonatal adiposity. The aim of the present study is to evaluate the effectiveness of a nutritional intervention based on encouraging the consumption of unprocessed and minimally processed foods and the practice of physical activities for appropriate weight gain in overweight, adult, pregnant women. Additionally, the effect of the intervention on pregnancy outcomes, neonatal adiposity, and the child’s weight and height will be investigated.

**Methods:**

This is a two-armed parallel randomized controlled trial that will be conducted at primary health units in Ribeirão Preto, SP, Brazil. Adult pregnant women who are overweight and receiving prenatal care in the public health system will be included. The women will be randomly allocated into control (standard care) or intervention groups. Those enrolled in the intervention group will participate in three individualized nutritional counselling sessions based on encouraging the consumption of unprocessed and minimally processed foods and the practice of physical activities. The recruitment of the participants will be carried out at seven health facilities over 12 months, with a sample of 300 women expected. Maternal anthropometric, sociodemographic, blood pressure, biochemical, and lifestyle data will be obtained at baseline (up to the 16th week of gestation), and during a second assessment (34th to 36th gestational week). The neonate body composition will be estimated after birth, and data on pregnancy outcomes, weight and height of children at 6, 12 and 24 months of age will be further obtained from medical records.

**Discussion:**

This will be the first randomized controlled trial to test the effectiveness of a nutritional intervention based on encouraging the consumption of unprocessed and minimally processed foods and the practice of physical activities for appropriate weight gain in adult, overweight, pregnant women. Furthermore, the effect of the intervention on pregnancy outcomes, neonatal adiposity and the child’s weight and height will be evaluated.

**Trial registration:**

Registro Brasileiro de Ensaios Clínicos (Rebec) RBR-2w9bhc July 30th 2018 (http://www.ensaiosclinicos.gov.br/rg/?q=RBR-2w9bhc+), and RBR-7yx36h June 4th 2019 (http://www.ensaiosclinicos.gov.br/rg/?q=RBR-7yx36h+0.

## Background

The high prevalence of overweight women of reproductive age is an alarming public health problem and may impact the health of the next two generations [1]. In addition to the immediate implications related to pregnancy complications, the pregnant woman being overweight can be a determining factor in the health of the child, being directly associated with the risk of developing obesity, cardiovascular disease, type 2 diabetes mellitus (DM) and asthma in adulthood [2].

Excessive weight gain during pregnancy exposes the mother-child binomial to the increased risk of deleterious health outcomes, such as macrosomia and neonatal hypoglycaemia, and hypertensive disorders, gestational diabetes mellitus (GDM), and postpartum weight retention in the woman [3–6]. Therefore, it is recommended that sustainable and effective life-cycle actions be taken to prevent chronic diseases [1].

Overweight women have a higher risk of excessive weight gain during pregnancy [7], probably due to the fact that overweight women are less likely to follow recommendations of healthy behaviour during the pregnancy [8].

In a meta-analysis study that included 65 randomized controlled trials that tested the effectiveness of dietary and/or exercise interventions on excessive weight gain in pregnant women, the authors concluded that there was sufficient evidence that these measures are effective in preventing weight gain in eutrophic women in the pre-gestational period, however, the strategies tested had limited efficacy among overweight women [9].

Contradicting these findings, the Lifestyle Interventions for Expectant Moms (LIFE-Moms) Consortium presented promising results in a meta-analysis of seven randomized controlled trials conducted among overweight and obese, American, pregnant women. This study showed that pregnant women in the intervention group had a 48% lower risk of gaining excessive weight when compared to the control group [10]. However, the number of investigations conducted in low and medium developed countries is considered insufficient, and the impact of intervention measures, adapted to the usual conditions of public health systems in these countries, for appropriate weight gain during pregnancy is unknown [9].

Recent evidence suggests the importance of investigating the relationship between food consumption and health outcomes taking industrial food processing into consideration. The NOVA food classification system classifies food according to the nature, extent, and purpose of processing. Foods are categorized into four groups: unprocessed or minimally processed foods, processed culinary ingredients, processed foods, and ultra-processed food and drink products [11, 12].

Increasing the consumption of ultra-processed foods [products are made by the food industry using substances extracted from foods or obtained through chemical syntheses (i.e., soft drinks, sugar-sweetened beverages, crackers, cookies, instant noodles, flavoured yogurts, and bread with additives)] to the detriment of meal-based eating patterns consisting of fresh foods [foods that have not undergone any industrial processing (i.e., fresh fruits, beans, and fresh meats)] or minimally processed foods [foods that were processed, however, without substances added or elements removed (i.e., coffee, natural fruit juices, and pasteurized whole milk)], is considered one of the factors related to the exponential global increase in the prevalence of obesity [11, 12].

Observational studies conducted in different geographic regions suggest a direct relationship between the consumption of ultra-processed foods and obesity in adults [13–17]. In Brazilian pregnant women, it was observed that women classified in the highest tertile energy percentage (%E) from minimally processed foods had a 51% lower chance of obesity, when compared to those with lower consumption. On the other hand, a positive association between higher %E from ultra-processed foods and obesity was found [3.06 (1.27–3.37)], regardless of confounding factors [18]. In a cohort of US pregnant women, it was found that each 1% increase in %E from ultra-processed diets was associated with a 1.33 kg increase in gestational weight gain and 0.62 percentage points in neonatal adiposity [19].

Ultra-processed products have low nutritional quality, high energy density and are rich in sugar, fat and salt, characteristics that make them hyperpalatable, resulting in impaired regulation of the appetite [12, 20]. In contrast, a higher intake of unprocessed or minimally processed foods is considered a marker for the consumption of handmade meals, prepared using nutrient-balanced and satiating foods, leading to a lower chance of obesity [12, 20].

An inpatient randomized controlled cross-over trial of ad libitum food intake conducted among American adults evaluated the effect of the consumption of ultra-processed products when compared to diets composed of unprocessed foods. The mean weight gain during the 2 weeks of ultra-processed diet was 0.9 (± 0.3) kg. Conversely, during the 2 weeks exposed to the diet based on unprocessed foods, the individuals lost a mean of 0.9 (± 0.3) kg, confirming the hypothesis of the deleterious effect of the consumption of ultra-processed foods on body weight control [21]. However, the effectiveness of nutritional counselling based on encouraging the consumption of fresh or minimally processed foods for weight control in free living individuals is not known.

Data from a meta-analysis that included 23 randomized controlled trials showed that physical activities interventions in pregnant women can reduce maternal gestational weight gain, especially for those with exercise frequency of three times per week and duration of 30 to 45 min. The practice of physical activity might attenuate insulin resistance and act directly on maternal metabolism, reducing the risk of excessive weight gain [22].

This is a two-armed parallel randomized controlled trial conducted with the aim to evaluate the effectiveness of a nutritional intervention based on encouraging the consumption of minimally processed foods and the practice of physical activity for appropriate weight gain in overweight, adult, pregnant women receiving prenatal care in primary healthcare units of the public health system in a Brazilian city.

The primary outcome of the study is appropriate maternal weight gain. Secondary outcomes are: mean weekly weight gain, changes in dietary intake and physical activity, changes in the biochemical profile of the pregnant women (fasting glucose, fasting insulin, total cholesterol and cholesterol fractions, triglycerides and C-reactive protein), complications in the pregnancy (gestational diabetes mellitus, hypertensive disorders and prematurity), birth weight, neonatal adiposity, and weight and length/height of the children at 6, 12 and 24 months of age.

## Methods/design

### Study setting

This is a two-armed parallel randomized controlled trial that will be conducted among overweight, pregnant women receiving prenatal care in seven Primary Health Units of Ribeirão Preto, SP, Brazil: Waldemar Barnsley Pessoa (Parque Ribeirão), Dr. Marco Antônio Sahão (Vila Virgínia), Dr. Sérgio Botelho da Costa Moraes (Jardim Presidente Dutra), Adalberto Texeira Andrade (Vila Recreio), Carlos Chagas (Vila Abranches), Rubens Issa Halak (Jardim Juliana) and Dr. Ítalo Baruffi (Castelo Branco). The Primary Health Units were selected according to the high demand for prenatal care and the minimum infrastructure necessary to conduct the study.

The study has been approved by the Research Ethics Committee of the School Health Centre of the Ribeirão Preto Medical School, University of São Paulo. Ribeirão Preto, SP, Brazil, (69,997,717.6.0000.5414, and 97,288,818.0.0000.5414).

### Eligibility and recruitment

Pregnant women who have started prenatal care at the Health Units selected will be identified regarding their eligibility potential through access to the information system of the municipal Health Department (*HygiaWeb*). Those potentially eligible will be approached by the interviewer at the time of the prenatal consultation. Women aged ≥18 years, gestational age (GA) up to 15 weeks and 6 days, pre-gestational body mass index (BMI) between 25.0 and 29.9 kg/m2 will be included. Pregnant women who report previous diabetes (or use of oral hypoglycaemic medication and/or insulin) and use of weight loss medications will be excluded. The original study protocol intended to exclude women in twin pregnancies, however, at the time of screening most of them will not have had access to the first ultrasound result.

### Interventions

The pregnant women that agree to participate in the study by signing the consent form will be randomly allocated to the intervention or control groups. The women allocated to the intervention group, in addition to the usual prenatal care at the Health Units, will be invited to participate in three individualized nutritional counselling sessions. Each session will last approximately 30 min. The counselling sessions will be held on days of return for medical consultations during the prenatal period, facilitating adherence and minimizing losses.

In the first meeting of the intervention all the pregnant women will be informed about the goals of the nutritional strategy (appropriate weight gain, daily consumption of fresh or minimally processed foods and regular practice of 150 min of physical activities per week), adopting the recommendations of the Institute of Medicine [23], Food Guide for the Brazilian Population [24] and American College of Obstetricians and Gynecologists [25] as the theoretical reference. It should be emphasized that pregnant women that present obstetric contraindications for the practice of physical activities will be instructed to follow the instructions of their physicians.

For the nutritional intervention strategy, educational material will be used consisting of three folders, one for each meeting, with key messages and illustrative images related to the goals set. All topics of the intervention will be addressed in the three meetings, although different approaches will be employed, appropriate for the gestational evolution. The development and validation of the educational material have been conducted prior to the implementation of the study.

Three folders were developed by the research team containing about six key messages and photos, encouraging appropriate weight gain, the consumption of fresh or minimally processed foods and the practice of physical activities. Intervention scripts were also developed as support material for the nutritionists responsible for conducting the intervention. For the semantic validation of the material, focus groups were conducted with pregnant women users of Primary Health Units, as well as nutritionists with professional experience in nutritional counselling.

The validation of the educational material consisted of the evaluation, adjustment and confirmation of the understanding of the instrument’s messages and images [26, 27]. The focus groups for the validation of the folders investigated whether there was relevance between the text and the illustrations; whether the language was understandable, accessible, clear and appropriate; whether the educational goals could be achieved with the messages and images; the practical feasibility of the proposed messages; whether the content was motivating and stimulated reflection; and whether the pregnant women identified with the messages and images. The focus groups for the scripts verified whether there was relevance between the message of the folder and the way it should be presented, whether the language was understandable, accessible, clear and appropriate; whether the educational objectives were achieved with the form of guidance: whether the proposed guidance was appropriate; and whether the content was motivating and stimulated reflection.

The original study protocol predicted that nutritional counselling sessions would take place at predetermined gestation periods: with the first meeting being conducted up to the 19th gestational week (GW), the second meeting between the 20th and 26th GW, and the third meeting between the 27th and the 33rd GW. However, due to the high rate of absenteeism observed in the prenatal consultations of the women included in the study, it was decided to make the intervention period more flexible, maintaining the three sessions between the first and second study evaluations.

The women allocated to the control group will receive only the usual prenatal care from the Health Units. After the birth, the participants in both groups will receive standardized nutritional counselling to assist them in recovering their pre-gestational weight.

### Outcomes

The primary outcome of the study is adequacy of weight gain. Weight measurements will be performed by trained nutritionists in the first and second study evaluations using a portable digital scale (Tanita®, model HS 302).

The measurement made by the nutritionists up to the 13th GW will be considered as the pre-gestational weight. For the women in the 14th GW included in the study, a value of 0.45 kg will be subtracted from the measured weight and for the women in the 15th GW included in the study a value of 0.91 kg will be subtracted from their measured weight. The results of these calculations will be adopted as an estimate of pre-gestational weight. The weight measured in the second study evaluation, preferably between the 34th and 36th GW, will be considered for the calculation of weight gain, rather than the weight measured at the moment of delivery. These procedures will be adopted due to the limitations regarding the use of self-reported pre-gestational weight and due to the high variability of water retention in the late gestation period, as well as the variability of the gestational age at delivery [28].

Weight data will be obtained from all the prenatal consultations. The mean weekly weight gain will be estimated through the difference between the weight measured in the second evaluation and the first weight recorded in the second trimester of pregnancy, divided by the number of days between the evaluation dates, divided by 7. The women with mean weekly weight gain between 0.23 and 0.33 kg in the second and third trimesters of the pregnancy will be considered to present appropriate weight gain. Insufficient weight gain will be considered for those with a mean weekly weight gain of less than 0.22 kg and excessive weight gain for women with weekly weight gain of more than 0.34 kg [23].

Secondary outcomes will be: mean weekly weight gain, changes in food intake, physical activity and the biochemical profile of the pregnant women (fasting glucose, fasting insulin, total cholesterol and fractions, triglycerides and C-reactive protein), complications in the pregnancy (gestational diabetes mellitus, hypertensive disorders and prematurity), birth weight, neonatal adiposity, weight and length/height of children at 6, 12 and 24 months of age.

Dietary intake will be assessed at the first and second study evaluations through two 24-h recalls on non-consecutive days in each assessment. The multiple-pass technique, performed by trained nutritionists, will be employed to obtain the recalls [29]. The Nutrition Data System for Research software, developed by the University of Minnesota in the United States, will be used to estimate the nutrients intake. Estimation of the usual diet will be carried out using the Multiple Source Method (MSM), a statistical modeling technique program developed by the European Prospective Investigation into Cancer and Nutrition (EPIC) [30]. The MSM eliminates the need for a high number of replications of the method [31] and has been found to be adequate for estimating the diet of pregnant women [32]. In addition, in the first and second evaluations of the study, the women will respond to a questionnaire on the frequency of the weekly consumption of foods of interest to assess adherence to the nutritional counselling program. The instrument used was adapted from the Surveillance System for Risk and Protection Factors for Chronic Diseases by Telephone Survey (*Sistema de Vigilância de Fatores de Risco e Proteção para Doenças Crônicas por Inquérito Telefônico* - VIGITEL) [33].

The practice of walking for leisure, walking for locomotion, and physical exercise will be assessed in the first and second study evaluations using a questionnaire that includes information on performance and duration in the week prior to the interview, according to the questionnaire adapted from the VIGITEL [33].

Blood samples from the pregnant women will be obtained in the first and second study evaluations after 8–12 h of fasting. Blood collection will be performed by a trained professional of the Health Units, by prior appointment. Fasting serum glucose and insulin levels, total cholesterol, LDL, HDL, triglycerides, and C-reactive protein will be measured. Blood glucose and lipid profile will be determined using enzymatic methods, insulin through the immunofluorometric method, and C-reactive protein using the chemiluminescence method.

Blood pressure will be measured in both study evaluations with an automatic digital arm blood pressure monitor (OMRON®, model HBP 1100, Omron Health Care Inc.), using the mean of three measurements in each evaluation. Pre-gestational hypertension will be considered if the woman presents blood pressure values greater than 140/90 mmHg in the first study evaluation, or if they report the use of antihypertensive medication at the time of the first study evaluation. Those without pre-gestational hypertension that present changes in blood pressure or are using hypertensive drugs at the time of the second study evaluation will be classified as having gestational hypertension [34].

Data for fasting glucose and at one and 2 hours after 75 g glucose load at the time of gestational diabetes mellitus (GDM) screening will be obtained from the *HygiaWeb* System. For the diagnosis of GDM the criteria proposed by the World Health Organization will be adopted, which require alterations in at least one glycemic value at any time during the pregnancy: from 92 to 125 mg/dL fasting, ≥180 mg/dL 1 h after glucose load or from 153 to 200 mg/dL 2 h after glucose load [35].

The third evaluation of the study will preferably be performed at the time of the Heel Prick Test, between the third to the fifth day of life of the newborn. If this is not possible, the third evaluation will be conducted at the postpartum consultation (maximum 10 days postpartum) or at the first childcare consultation (within 15 days of life). Using a structured questionnaire, the following variables will be obtained: number of prenatal visits to the Health Unit, continuous use of medications during the pregnancy, report of chronic health problems of the puerpera, date and type of delivery, gestational age at delivery, maternal complications (hypertensive disorders and hyperglycaemia), and complications before, during and after the birth concerning the mother and the infant. Anthropometric measurements of the baby at birth will be obtained from the maternity ward discharge form.

The newborn’s body adiposity will be measured in the third evaluation of the newborn using anthropometric measurements according to standardized procedures [35–38]. The estimation of the newborn’s body adiposity will be performed using two distinct models, one proposed by Catalano et al. [39, 40] and the other proposed by Deierlein et al. [41]. These models were validated against the air displacement plethysmography (PEA POD®) method, with data accuracy estimated at 83 and 81%, respectively.

### Participants timeline

**Fig. 1:**
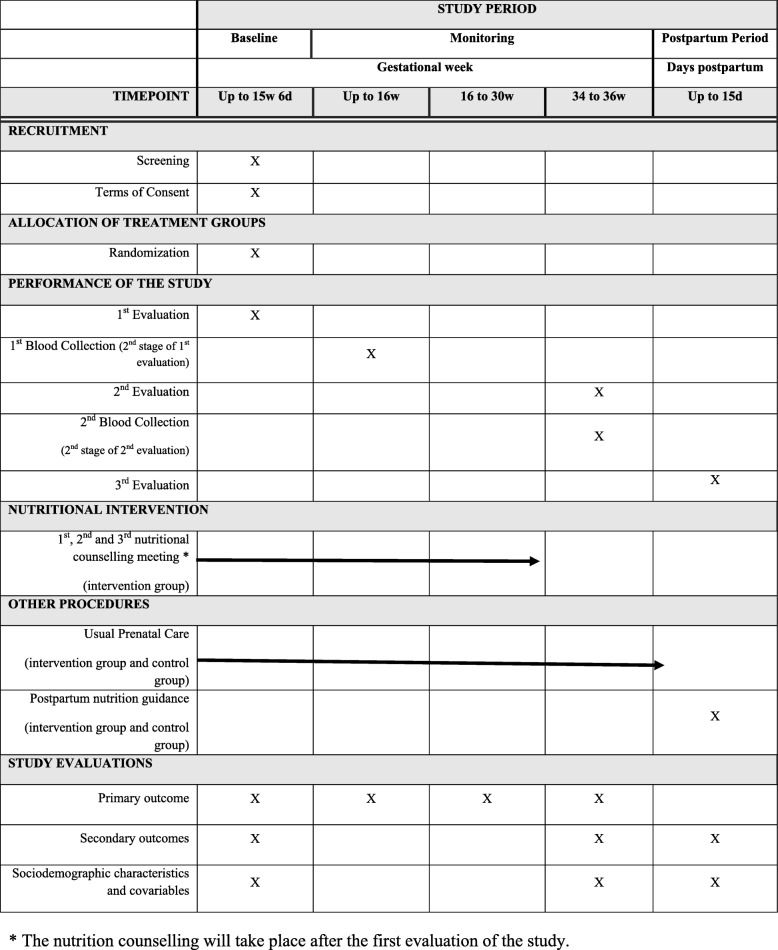
Sequence of recruitment, intervention, and evaluation activities of the study participants. * The nutrition counselling will take place after the first evaluation of the study.

Figure 1 presents an outline of the sequence of the recruitment, intervention, and evaluation activities of the study participants.

### Sample size

The sample size calculation was based on the primary outcome of the study. A 20% difference in the proportion of adequacy of weight gain between the treatment groups is expected [42]. Considering a minimum significance level of 5% (α = 0.05) and a power of 90% (β = 0.1), with a 20% loss during monitoring, a sample of 300 women will be sufficient.

### Allocation

The eligible pregnant women that agree to participate in the study will be randomly allocated to the treatment groups. Randomization will be performed directly using the Research Electronic Data Capture (REDCap) software [43, 44], with a spreadsheet of numbers randomly generated by Microsoft Office Excel. Stratification between the groups will be performed considering the prenatal care Health Unit, which may differ in relation to the quality of the prenatal care offered and the socioeconomic profile of the users. Given the nature of the intervention, both the study participants and the researchers will be aware of the group allocation.

### Data collection methods

The pregnant women of both groups will undergo two evaluations in the Health Units during the pregnancy, at the time of the usual prenatal consultation. The first assessment (baseline) will be conducted prior to the 16th gestational week (GW) and the second assessment will preferably take place between the 34th and 36th GW. The third evaluation of the study comprises the analysis of the mother-child binomial. The approach in the third evaluation of the study will preferably be made at the time of the Heel Prick Test, between the third and fifth day of life of the newborn. If this is not possible, the third evaluation will be conducted at the postpartum consultation (maximum 10 days postpartum) or at the first childcare consultation (within 15 days of life). In addition, secondary data on the puerpera and the newborn will be collected from electronic medical records (*SisprenatalWeb*, *Floresce uma Vida* and *HygiaWeb*).

Study data will be collected and managed using the REDCap electronic data capture tools hosted at the Ribeirão Preto Medical School, University of São Paulo [43, 44]. REDCap is a secure, web-based software platform designed to support data capture for research studies, providing 1) an intuitive interface for validated data capture; 2) audit trails for tracking data manipulation and export procedures; 3) automated export procedures for seamless data downloads to common statistical packages; and 4) procedures for data integration and interoperability with external sources.

### Data management

To ensure the quality of the data collected, the research group will consist of trained nutritionists. A guidance manual has been prepared, prior to data collection, to be used as a protocol for all study procedures. It should be emphasized that the REDCap system [43, 44] has mechanisms called “bounds” that aim to reduce possible errors in the data collection step. In addition, all information collected will be checked by a researcher other than the one that entered the data into the system.

### Statistical analyses

The analyses of this study will follow the intention to treat principles. The homogeneity of the sample at the beginning of the study will be verified using Student’s t-test for independent variables or the Mann-Whitney U test, depending on the data distribution, for the continuous variables; and the X^2^ test for categorical variables. For the continuous variables the delta will be calculated and differences with the baseline will be tested using Student’s t-test for dependent variables or the Wilcoxon test. The impact of the intervention on the proportion of women with appropriate weight gain will be assessed by means of the relative risk and 95%CI adjusted for confounding factors.

The statistical correlation method will be used to investigate the impact of the intervention on neonatal adiposity and birth weight. If there is linearity between the treatment group data and outcomes, linear regression models will be employed, adopting adiposity and birth weight as the dependent variables, adjusted for potential confounders.

The analyses will be conducted using the SPSS® software (version 24) and *p* values < 0.05 will be considered significant.

## Discussion

This clinical trial will be unprecedented due to investigating the effectiveness of a nutritional intervention strategy based on encouraging the consumption of fresh or minimally processed foods and physical activity in overweight, pregnant women for appropriate weight gain and neonatal adiposity. Additional investigations will include changes in dietary intake and physical activity, changes in the biochemical profile of the pregnant women (fasting glucose, fasting insulin, total cholesterol, cholesterol fractions, triglycerides and C-reactive protein), pregnancy complications (GDM, hypertensive disorders and prematurity), birth weight, and weight and length/height of the children at 6, 12 and 24 months of age.

Although promising results regarding appropriate weight gain in overweight, pregnant women have recently been demonstrated by LIFE-MOMs [10], the number of investigations into the impact of intervention measures adapted to the usual conditions of public health systems of countries of low and medium development is still considered insufficient [9]. Furthermore, increasing evidence from observational studies suggests deleterious effects on the health of adults and pregnant women due to the high consumption of ultra-processed products [13–19].

Only one previous clinical trial investigated the effect of unprocessed food consumption on weight control in adults [21]. In the study by Hall and colleagues [21], all meals were provided to the study participants, who did not face obstacles related to food acquisition and preparation, steps necessary to adopt a diet based on the consumption of fresh or minimally processed food. In addition, ultra-processed products are widely offered through various marketing and advertising strategies, which influence the food choices of free living individual [12, 20]. Accordingly, the present study could advance our knowledge regarding the effectiveness of such a strategy.

The fact that the study is unprecedented and the feasibility of the implantation of the strategy in public health services of countries of low and medium development can be considered important strengths. In addition, all the assessments will be made by trained nutritionists to ensure the quality control of the data. As all the study evaluations will be conducted in Primary Care Units, the evaluation of some newborns will be conducted after 72 h, which may affect the estimation of adiposity. To minimize this limitation, age-adjusted models will be employed [41]. We believe the results of the study will expand the knowledge regarding the effect on maternal and child health of an intervention based on the degree of food processing and on encouraging the practice of physical activity.

## Data Availability

Datasets arising from the study might be available upon reasonable request from the corresponding author: daniss@fmrp.usp.br

## Abbreviations

BMI: Body mass index
DM: type 2 diabetes mellitus
GDM: Gestational diabetes mellitus
GW: Gestational week
GWG: Gestational weight gain
MSM: Multiple source method
REDCap: Research Electronic Data Capture
VIGITEL: *Sistema de Vigilância de Fatores de Risco e Proteção para Doenças Crônicas por Inquérito Telefônico*
